# Kaposi Sarcoma in the United States: Understanding Disparate Risk

**DOI:** 10.1093/jncics/pkac079

**Published:** 2022-11-10

**Authors:** Anne S Reiner, Katherine S Panageas

**Affiliations:** Department of Epidemiology and Biostatistics, Memorial Sloan Kettering Cancer Center, New York, NY, USA; Department of Epidemiology and Biostatistics, Memorial Sloan Kettering Cancer Center, New York, NY, USA

The epidemiologic and medical communities have witnessed a parallel story of HIV/AIDS and Kaposi Sarcoma (KS) unfold for the better part of 4 decades. Prior to the 1980s, KS was rare in the United States (1). Following the onset of the HIV/AIDS epidemic, incidence of KS rose steeply (2) and the two became inextricably linked with KS becoming an AIDS-defining condition (3).

In the 1990s, KS incidence sharply declined, which was attributed to a decrease in HIV/AIDS-related immunosuppression due to both the introduction of antiretroviral therapy (4) and how the community of men who have sex with men (MSM) responded to the HIV/AIDS epidemic (5), reducing the number of unprotected sex acts with new partners, the latter altering the transmission of HIV (6).

Recently, more nuanced trends of HIV and KS have emerged relating to age, sex, race, and geography. Overall, both HIV (7) and KS (8) incidence have been decreasing. However, incidence has been increasing for subgroup populations. Studies of HIV infections have shown that although rates are decreasing for most transmission groups, young men, particularly MSM (7) and men in the South (9), are experiencing increases in HIV incidence. Similarly, although KS incidence in persons living with HIV is decreasing (8), the literature is beginning to reveal subpopulations where this is not borne out. Incidence is stable for young, Black persons living with HIV (8), and incidence is rising for Black men living in the South (10). In this issue of *JNCICS*, Suk et al. (11) further elucidate who is most at risk of KS, an issue of critical importance for targeting HIV prevention, early HIV diagnosis, and early HIV treatment.

In their article (11), Suk and colleagues use the National Program of Cancer Registries (NPCR) (12) and Surveillance, Epidemiology, and End Results (SEER) (13) Program Database, which provide deidentified, population-based cancer incidence data across the entire US population. The NPCR and SEER Program are surveillance systems well suited to investigate several facets of cancer not limited to incidence, prevalence, survival, temporal trends, and subgroup patterns, all with the goal of advancing epidemiologic knowledge and informing clinical inquiries. Since its inception in the early 1970s, SEER has broadened geographical coverage over time and is considered the gold standard in rigorous, high-quality data collection (14). Coupled with the NPCR, which was established in 1992, these nationally representative, rich data sources are uniquely positioned to investigate who is at highest risk for specific cancers like KS so that health-care resources can be efficiently allocated for those who most require it.

From 2001 to 2018, Suk et al. (11) reported using NPCR and SEER data showing that the KS incidence rate statistically significantly decreased by 3.5% per year in young, non-Hispanic White men but statistically significantly increased 1.5% per year in young, non-Hispanic Black men, among whom, those living in the South experienced a statistically significant 3.3% per year increase in KS incidence whereas their non-Southern counterparts experienced no change.

To complement these results, Suk et al. (11) examined the birth cohort effect across 16 birth cohorts to more deeply understand the time trends for KS in young, non-Hispanic Black men using age-period-cohort (APC) analysis. APC can be indispensable in the epidemiologic interrogation of longitudinal data (15). In particular, cancer registries follow a cohort of cohorts, and using APC can illuminate changes that are not always detectable with other epidemiologic study designs (16). Among non-Hispanic Black men in the South, those born in recent years had a twofold higher risk of KS compared with those born in earlier years. This was not true of non-Hispanic Black men in non-Southern regions, where no birth cohort differences were observed (11).

Suk and colleagues (11) have identified a very specific, targetable subpopulation at highest risk of Kaposi Sarcoma: young, non-Hispanic Black men in the Southern United States, particularly those born in more recent years. Although NPCR and SEER do not collect information on HIV or AIDS, we can make some reasonable inferences based on prior observations from the literature. Because approximately 90% of KS cases in men are concomitantly in men with HIV/AIDS (17) and because it has also been shown that HIV incidence has increased in young, non-Hispanic Black MSM in the South (9), it is likely that the observed increase in KS shown by Suk et al. (11) is caused by rising HIV rates, both diagnosed and undiagnosed (7). Moreover, in general, Southern states have larger proportions (18) of uninsured people, and uninsured rates are also higher for Black people than White people (19), which inevitably affects health-care use. And, notably, Black MSM have been shown to have less HIV testing, more undiagnosed HIV, and later diagnosed HIV, all associated with structural health-care service and societal barriers such as HIV-related stigma, discrimination, and poverty, which occur at high levels in the South, particularly in resource-poor locales (20).

The contribution of HIV diagnoses in people who inject drugs cannot be discounted. Of the HIV diagnoses attributed to drug injection in 2018, the Southern US contributed to almost 40% (21). The opioid epidemic, which has quintupled drug overdose deaths since 1999 (22), has also incited an increase in diseases associated with injection drug use (23). Community-based syringe services programs (SSPs) are successful in providing counseling, testing, and sterile injectors and allowing for the safe disposal of used injectors (24). People who use SSPs are threefold more likely to stop using drugs and fivefold more likely to enter drug treatment than people who do not use SSPs (23). During the study by Suk et al. (11), in 2008, there were several Southern states that did not have a single SSP statewide, including Georgia, Alabama, Mississippi, South Carolina, Tennessee, and Kentucky (25). Even today, some Southern states do not have state laws removing barriers to the legality of SSPs, including Alabama and Mississippi (26). In general, SSPs are least likely to be located in rural areas and Southern states (27,28).

Aggregate incidence trends of HIV and KS portray an encouraging narrative, but Suk et al. (11) are to be congratulated for identifying a subpopulation on whom the medical and general community must focus. Outreach and education efforts can be facilitated through local leaders, including in the medical, business, and religious communities. HIV prevention, additional and well-placed SSPs, better and early HIV diagnosis, adequate treatment, and improved access to treatment are imperative for young, non-Hispanic Black men in the United States South, a subpopulation for whom there also exists considerable outcome disparities (10,20).

## Funding

This work was supported by the US National Institutes of Health (P30 CA008748).

## Notes

**Role of the funder:** The funder had no role in this editorial.

**Disclosures:** The authors have no disclosures.

**Author contributions:** Conceptualization, writing—original draft, writing—review and editing: **ASR, KSP**.

## Data Availability

No data were generated or analyzed for this editorial.
